# Optimising delivery of integrated palliative care and heart failure services: A realist evaluation protocol (PalliatHeartSynthesis II)

**DOI:** 10.1371/journal.pone.0341418

**Published:** 2026-02-18

**Authors:** Tracey McConnell, David Scott, Geoffrey Wong, Loreena Hill, Miriam J. Johnson, Yvonne Millerick, Joy Ross, Clea Atkinson, Linda Cooper, John Burden, Rachel Campbell, Andrew Kerr, Honey Thomas, Joanne Reid

**Affiliations:** 1 School of Nursing and Midwifery, Queen’s University Belfast, Belfast, United Kingdom; 2 Nuffield Department of Primary Care Health Sciences, University of Oxford, Oxford, United Kingdom; 3 School of Nursing and Paramedic Science, Ulster University, Londonderry, United Kingdom; 4 Wolfson Palliative Care Research Centre, Hull York Medical School, University of Hull, Hull, United Kingdom; 5 Glasgow Royal Infirmary and Glasgow Caledonian University, Glasgow, United Kingdom; 6 St Christopher’s Hospice, Sydenham, London, United Kingdom; 7 Palliative and Supportive Care Department, Cardiff and Vale University Health Board, Cardiff, United Kingdom; 8 Patient and Public Involvement, Independent Representative, Belfast, Northern Ireland; 9 Patient and Public Involvement Network members, British Heart Foundation, London, United Kingdom; 10 South Eastern Health and Social Care Trust, Dundonald, Belfast, United Kingdom; 11 Northumbria Healthcare NHS Foundation Trust, Newcastle Upon Tyne, United Kingdom; PLOS: Public Library of Science, UNITED STATES OF AMERICA

## Abstract

**Background:**

Inequality in palliative care provision is an ongoing problem for underserved groups, such as those with heart failure (HF) placing a burden on patients, their caregivers and health services due to frequent, often avoidable hospitalisations. Our realist synthesis of integrated palliative care (PC) and HF literature found that integrated HF and PC services work best when service providers are motivated and have the opportunity and capacity to support behaviour change. However, we identified significant knowledge gaps with most studies completed in United States of America (USA) and based primarily on the views of nurses and physicians. We developed strategies to help services provide integrated PC and HF services but identified the need for United Kingdom (UK) primary data to better understand the context-specific implementation of palliative care and HF care.

**Methods:**

This project will use co-design and realist evaluation to generate data from five PC and HF integrated services in the UK, purposively sampled to provide variation in geography and service design (Research Registry 10624). The research comprises three work packages (WPs). WP1 will deliver a realist evaluation of each site including documentary analysis, observations and semi-structured interviews with service providers and users. WP2 uses co-design methods to develop a guide to help others set up and improve integrated PC and HF services. Data from both WPs will be analysed and synthesised using a realist logic of analysis. WP3 will facilitate the development of a community of practice to support those who wish to set up, sustain and embed integrated PC and HF services.

**Discussion:**

This realist evaluation of a complex intervention will improve understanding of how to tailor and implement integrated PC and HF services. The co-designed ‘how-to guide’ and community of practice will facilitate knowledge translation and ensure that evidence-based guidance is provided to assist in service development.

## Background

Heart failure (HF) has been described as a modern-day global epidemic which is increasingly common among aging populations [1,2]. HF is associated with multiple long-term conditions, and this debilitating disease is expected to affect 64 million people worldwide by 2050 [2]. The absolute number of people with HF in the UK during 2014 was 920, 616 (1.4% of the overall population), outnumbering patients with the four leading forms of cancer combined [3]. Patients and their informal caregivers (carers) experience a range of debilitating physical, psychological and social symptoms which can lead to high dependency on healthcare services and repeated hospital admissions, especially during the advanced stages of the illness including the patient’s last year of life [4]. In total HF costs the National Health Service (NHS) within the UK an estimated £2bn per annum [4]. In 2014, the global economic burden of HF was estimated at over £80 billon per annum [5].

The timely provision of integrated Palliative Care (PC) and HF management, offers an effective and cost-effective response to many of these issues by helping to relieve suffering, improving the quality of life of both patients and carers, reducing days in hospital and health service costs [6,7]. A literature review published in 2016 summarised the evolving role of PC for patients with HF along with the barriers and opportunities for its integration into routine practice [8]. Findings from this review highlighted the need to develop evidence on how best to integrate PC and HF services given the cultural and environmental differences in how PC services are delivered. Since then, three further systematic reviews have been published [9–11], providing further evidence on the effectiveness of multi-component, multi-disciplinary integrated PC and HF interventions for improving patient-centred outcomes such as symptom burden and quality of life, and reducing healthcare resource utilisation with no impact on survival rates. The integration of PC services is also recommended in HF clinical guidelines globally [12–14]. However, two decades after the first calls to redress the lack of support, planning and holistic care available to those living with and dying from HF, and despite evidence supporting the use of multidisciplinary integrated PC, people with HF remain less likely to receive PC or may receive it later in their disease trajectory than people with cancer [9,15,16].

Integrated PC and HF services aim to achieve continuity of care by integrating administrative, organisational and clinical services that make up the care network [17]. Integrated PC and HF interventions should comprise cohesive working across all care settings. Additionally, such interventions should be underpinned by unified and consistent communication alongside simultaneous HF and PC, including end of life care management as appropriate, to improve person-centred outcomes [12–14]. The available evidence demonstrates that, although there are examples of good practice [9,15], these are not routinely implemented, and there is substantial variation in the delivery of services to patients and carers. Inequity of PC access is evident and the PC needs of people with HF have often been overlooked resulting in calls for more attention to, and research for, this vulnerable group [6–9,13,15].

**Fig 1:**
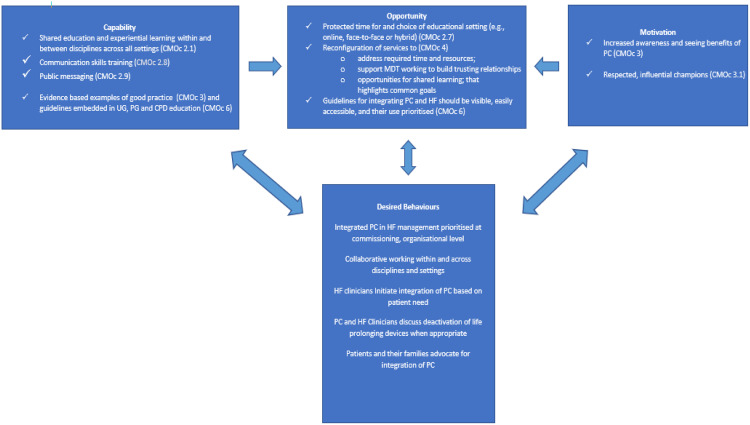
PalliatHeartSynthesis Programme Theory.

There is a need to understand why the integration of PC into HF services has been slow and inconsistent. Realist approaches seek to answer the question what works, for whom, in what circumstances and why? [18,19]. As part of an ongoing programme of research, our team completed a realist synthesis of the literature to understand why PC has, or has not, been routinely integrated into HF services and developed implications for policy and practice [20,21]. The realist analysis developed six overarching context-mechanism-outcome configurations (CMOcs) with 30 sub-CMOcs. The resulting programme theory was summarised in relation to the three core components of the COM-B model – capability, motivation and opportunity. Findings were presented in this way as both the literature and stakeholder feedback indicated that the key barriers to integrating care largely involved human behaviour in response to underlying processes (motivation etc.) shaped by the contexts in which key players operate (Fig 1). Although the COM-B model relates to individual behaviour change, it also considers team and organisational behaviour. For example, an individual’s capacity to carry out a specific behaviour also depends on the organisational culture they work within, and the opportunities afforded to them within their team and organisation. Findings suggest that integrated PC and HF services work best when there is protected time for evidence-based education and choice of educational setting (e.g., online, face-to-face or hybrid). Other key intervention strategies included the importance of increased awareness of PC and seeing benefits of PC for those impacted by HF. Findings also highlighted the importance of credible champions for spreading the emotive and intellectual need for integrating PC, seeing direct patient benefit, and having visible guidelines for integration. Based on these findings a series of implications and strategies were developed for those working to set up integrated PC and HF services (Fig 1). The contents of Fig 1 will form the basis of the initial programme theory of this realist evaluation and will be developed and tested further.

The realist synthesis identified significant knowledge gaps. Most included documents were from the USA, with only 30 of the 130 included documents specific to the UK. Most documents considered the experiences of nurses and physicians only, with few including perspectives from the wider multidisciplinary team. There is therefore a need to undertake a realist evaluation, which collects and uses primary data, to enable us to further develop, confirm, refute, or refine our ‘implications’ to optimise the delivery of integrated palliative care and HF services. This realist evaluation aims to increase our understanding by addressing the following research question: ‘What can we learn from existing integrated PC and HF services across the UK, to develop a detailed ‘how to’ guide for setting up, sustaining, and embedding integrated HF and PC services’? Potential transferability of our findings to other settings will be explored.

## Aim

Realist methodology is increasingly used in the assessment of complex interventions [22] and is deemed the most likely method to deliver results that will help to inform policy makers and service planners in developing and delivering reconfigured services. The methodology will allow us to learn from existing successful integrated PC and HF services and help to develop detailed ‘how to’ guidance for setting up, sustaining, and embedding integrated PC and HF services.

### Objectives

1. Use a realist approach to evaluate existing integrated PC and HF services to understand how they have been set up, sustained, and embedded in routine practice.
2. Use these data to confirm, refute or refine our existing programme theory developed during our realist synthesis [21].
3. Use co-design methods to develop a ‘how to guide’ to help other services provide integrated PC and HF care.
4. Develop a community of practice to support those who wish to set up, sustain and embed integrated HF and PC services.

### Design and methods

#### Realist evaluation and co-design.

This project started in January 2025 and will be completed over three-stages by December 2027 using realist evaluation [22] and co-design [23] approaches. Participant recruitment and data collection have started at two sites, with data collection at the remaining three sites to be completed by June 2027. The results are expected by mid-2028, depending on publication timelines. Primary data will be generated at five sites to further develop the programme theory describing how best to set up and run integrated PC and HF services developed during the realist synthesis [21]. This will produce recommendations and consider issues relating to the implementation of these recommendations through the co-designed ‘how to’ guide. This research design is underpinned by MRC guidance for the development, evaluation, and implementation of complex interventions to improve health [24]. All data will be collected by the lead Research Fellow (DS). Ethical approval was provided by the South East Scotland Research Ethics Committee 02 (IRAS ID: 337143). All participants provide their written informed consent to participate in the research.

Co-design methods are based on a philosophy and ethic of equity. Taking a pragmatic approach, co-design methods enable the researcher to be responsive and bridge the gap between theory, research and practice enabling researchers and stakeholders to drive the research forward and refine the approach to answer the research question [24]. This partnership approach will challenge hierarchy within the research team and across stakeholders [25] and fits well with realist approaches.

Realist approaches assume that few (if any) programmes or interventions work everywhere for everyone: context makes a considerable difference to programme outcomes [26]. We have adopted a realist approach because it was clear from the realist review [21] that integrated PC and HF services are complex and how well they work (or not) depends on context, who delivers them and how. Furthermore, integrated PC and HF services typically do not really pre-exist as an ‘off the shelf’, product. Health services vary greatly depending on differences in geographical patterns of disease, clinicians’ behaviour, incentives in health care financing etc. This makes theory – driven realist approaches particularly suitable for this problem area [22].

Realist approaches identify causal processes (mechanisms) for outcomes of interest (intended and unintended). Findings are potentially transferable because in realist approaches the assumption is that most mechanisms are widely occurring in different settings, situations and/or people, but may not be activated unless the right contexts exist [18]. Using a realist evaluation approach will enable us to focus on the mechanisms (causal processes) for desired and undesired outcomes from integrating PC and HF services. Our focus on transferable causal mechanisms will enable us to make ‘recommendations’ for integrated PC and HF services that are likely relevant and applicable across different settings, including other health service models and jurisdictions.

### Sites and sampling

Five integrated UK-based PC and HF services were purposively sampled to involve specific groups of multidisciplinary integrated PC and HF service providers across all care settings (e.g., primary care, secondary care, third sector) ensuring diversity of the populations they serve, and varied service models.

Both sites in England (St. Christopher’s Hospice and Northumbria Healthcare NHS Foundation Trust) were selected due to their differences (large urban *versus* town/rural; north *versus* south; minority ethnic representations; model of PC-HF service delivery). We also included devolved nation sites in Scotland (NHS Greater Glasgow and Clyde), Wales (Cardiff and Vale University Health Board), and Northern Ireland (NI) (South Eastern Health and Social Care Trust) to ensure UK-wide relevance. We chose the sites in Scotland, Wales and NI as they provide additional variation in terms of their development and their degree of integration, to help provide an in-depth understanding of the integrated PC and HF process across primary and secondary care in the UK (see Table 1).

**Table 1:** Description of Study Sites.

Location	Participant Sample (inpatient & outpatient)	Population diversity
England site 1	- Cardiologist - PC Consultant - PC and HF nurses - District nurse - GP - Pharmacist - Admin support - Spiritual care/ Chaplaincy - Social worker - Complementary Therapist - Pacing clinic health care provider (deactivation of ICDs) - Commissioner - Patient - Informal carer	St Christopher’s Hospice serves a diverse population of 1.5 million in the London boroughs of Bromley, Croydon, Lambeth, Lewisham and Southwark reaching some of England’s most deprived areas. Bromley has largest frail older population of London Boroughs. The CCG and St Christophers jointly funded and evaluated a pilot heart failure/ palliative care service working across the hospital/community in Bromley and linked closely with local GPs. This includes all HF phenotypes; 1/3 of patients with preserved ejection fraction. The success of this venture supported successful application for external funding to extend and adapt/test a similar service in Croydon, which has a more ethnic diverse population and sits within a neighbouring ICS. Currently 25% of service users live alone.
England site 2	• Cardiologist • HF specialist nurse (secondary care) • Community HF specialist nurse • District nurse • Cardiac physiologist (pacing/ICD clinic care provider) • PC nurse • PC doctors • District nurse • Spiritual care/ Chaplaincy • GP • Patients and carers	Serving one of the largest geographical areas of any NHS trust in England, Northumbria Healthcare provides a wide range of services to more than half a million people living in Northumberland and North Tyneside. The Trust provides services to people that live in urban areas (North Tyneside and southeast Northumberland) and to those that live in some of the most rural parts of England. Both areas have industrial histories ranging from ship building to coal mining, with limited population movement out of area (creating an older age profile) and as such the local population has some of biggest health challenges nationally. There are high rates or cardiovascular disease and lower life expectancy than national averages.
Scotland	- Patient - Informal carer - Cardiologist - PC Consultant - COE Consultant - HFPC Nurse Consultant - PC/HF/ANP & DN nurses - GP - Pharmacist - Pharmacist Assistant - Admin support - Hospice Services - Cardiac Physiologist Department (Pacing/Echo/ECG/ deactivation of ICDs) - Long Term Conditions Benefits Services - Spiritual care/ Chaplaincy - Health & Social Care - OT/Physio/Dietician/Psychologist/ - Access to other services as appropriate.	Diverse & predominantly deprived geographical population. Inclusive of all HF phenotypes (HFrEF & HFpEF, Valvular patients not suitable for corrective intervention & Amyloidosis patients). Patients with CRT-D or ICD cardiac devices that requires timely deactivation. The service approach consists of clinic, telephone and/or home visiting to suit individual person focused needs. Care Home Patients are also included as these patients are predominantly excluded from the HF service across Glasgow. Patient referrals from the advanced transplant centre who reside within the North East Glasgow area will also be included although the numbers are low.
Wales	- Cardiologist - PC Consultant - Junior Doctors - PC and HF nurses - District nurse - Occupational therapist - Physio - Psychologist - Dietician - Aseptic Unit Team - Pharmacy Team - GP - Admin support - Spiritual care/ Chaplaincy - Value based project manager - Finance Team - Pacing clinic health care provider (deactivation of ICDs) - Commissioner - Patient - Informal carer	Cardiff and Vale UHB serves a population of 600 000 with a multi-cultural/multi-sociodemographic, and is both a secondary and tertiary centre including patients from a wide area across South West, East and Mid Wales with significant ethnic population mix and deprivation within the urban areas and wider reach to areas with significant rurality. C&VUHB is also the cardiothoracic surgical centre. Patient groups include: younger patients not suitable for transplant, patients with preserved and reduced ejection fraction, patients with valvular disease, patients with amyloidosis, patients with complex devices. Services are adapted for patients who can’t afford/unable to travel (e.g., clinics: including hot review, online appointments, phone support, home visits, hospital review (a cross-boundary model), access to ambulances, multidisciplinary team including occupational therapy, physiotherapy, dietetics and psychology as well as clinical nurse specialists and medics. The integrated PC and HF model in Wales covers urban and rural areas, with a community of practice forum. Referrals include patients from tertiary adult congenital heart disease services. The Welsh government have invested Value-Based recurrent funding for expansion of the integrated model to other chronic disease pilot areas.
Northern Ireland	-Consultant Cardiologist -PC Consultant - Cardiology Specialty Doctor - HF Specialist Nurse - PC Specialist Nurse - Community PC nurse - Spiritual care/ Chaplaincy - Admin Support	Lagan Valley Hospital is a local acute hospital with an urgent care centre which is part of the South Eastern Health and Social care Trust. It is an integrated Trust, incorporating acute hospital services, community health and social services. The newly established integrated service is inclusive of all heart failure phenotypes including HFrEF and HFpEF, patients with CRT-D or ICD in situ requiring device deactivation discussions and patients with frailty

To ensure research inclusion, we will work closely with our site leads and Patient and Public Involvement (PPI) co-applicant to ensure our service user sample is representative of HF populations, such as younger working age adults that are not suitable for transplant, older people living with multiple long-term conditions, those living in deprived areas, those with HF with preserved ejection fraction, those with complex devices, non-English speaking etc.

### Work package 1: Realist evaluation of existing integrated PC and HF services

WP1 will provide a detailed understanding of existing services in the UK that are currently delivering integrated PC and HF services. The primary data we collect will enable us to confirm, refute or refine parts of our existing programme theory [21] (see Fig 1). We will use the improved programme theory to update existing ‘policy and practice implications’ on how integrated PC and HF providers can reconfigure their existing services. Where relevant, we will draw on existing frameworks (e.g., behaviour change research by Michie [27,28]) to help us develop this ‘how to’ guidance.

A summary of data collection activities at each site is presented in Table 2. The evaluation will involve semi-structured interviews with up to 20 stakeholders, identified via purposive sampling (to ensure inclusion of participants who have insight into how integrated PC and HF services have been set up, sustained and embedded in routine practice – e.g., healthcare professionals (HCPs) n = 15, patients and/or informal carers n = 5 in total) at each of the five sites.

**Table 2:** Summary of Data Collection at each site.

Location	Documents (WP1)	Audit Data (WP1)	No. of Interviews (WP1)	Observations of integrated PC and HF services (WP1)	No. of Focus Groups (WP2)
England site 1	Site specific	Site specific	20	1-3	2
England site 2	Site specific	Site specific	20	1-3	2
Scotland	Site specific	Site specific	20	1-3	2
Wales	Site specific	Site specific	20	1-3	2
Northern Ireland	Site specific	Site specific	20	1-3	2
Total	--	--	100	5-15	10

Study participants will be people with heart failure who are receiving care from the integrated palliative and heart failure service, their primary family caregiver and healthcare professionals who are delivering the integrated service or are knowledgeable about the service (see Table 3). The final sample size will be determined when we have enough data for theoretical saturation [22]. Sampling for realist interviews is theory based, i.e., participants are selected because they can cast light on a hypothesis or a particular aspect of the programme theory [22]. Because the unit of analysis is not the person but the events and processes around them, every respondent can uncover a collection of micro events and processes, each of which can be explored in multiple ways to test theories. This means that a relatively small number of participants with detailed knowledge of the integrated service can be interviewed multiple times to confirm, refute or refine the developing programme theory [22].

**Table 3:** Inclusion criteria for participants.

Inclusion criteria	Screened by
**Patient**	
Has received a diagnosis of heart failure and is receiving care from the integrated palliative care and heart failure service.	Treating clinician or member of direct care team.
Over 18 years of age.	Research Team.
Written informed consent.	Research Team.
**Family caregiver**	
Primary informal caregiver as identified by the HF patient.	Patient.
Patient has consented to carer being approached.	Research Team.
Over 18 years of age.	Research Team.
Written informed consent.	Research Team.
**Healthcare Professional**	
A member of the Integrated Palliative Care and Heart Failure Service.	Member of the direct care team and Research Team.
Written informed consent.	Research Team.

Example interview questions are outlined below:

- How well is the palliative care and heart failure service working? What’s making it work well/ not so well?
- Do you think that the impacts have been the same for all [e.g., people living with heart failure, informal carer for someone living with heart failure, HCP overseeing/providing care to a person living with heart failure]?
- If you could change something about the way you manage heart failure patients and their palliative care needs to make it work more effectively here, what would you change and why?

We will also seek out any site-specific routine data from each site (e.g., reports, case summaries, audit data, and patient reported outcome measures (PROMs)) to shed light on what facilitated or hindered set up, sustaining and embedding of integrated PC and HF services. This will be supplemented with focused observations and field notes (n = 1–3 per site) of the integrated PC and HF services, to explore how teams and systems operate on the ground. A participatory ethnographic approach [29] will guide the observations of the service, from where patients park, distance to their heart failure appointment, right through to layout of consultation rooms, nature of service delivery discussions and use of IT equipment to support integration. Ethnography seeks to understand the culture of a particular setting or environment. It is an inherently co-constructed process of research practice that emerges and evolves over a period of sustained co-inquiry, rather than inquiry driven by the researcher’s interests. Observation and field notes are corner stones of the approach, which allow the development of relationships with research participants providing an insight into the context, with a focus on the culture and social interaction of the subject of study. Ethnography is particularly valuable in understanding the influence of social and cultural norms on the effectiveness of health interventions and understanding how complex interventions work. These ethnographic activities will enable us to ‘see’ if what we are told plays out as expected in practice.

### Data analysis of observations, site specific data, and interviews

All field notes from the observations, site specific data and interview transcripts will be entered into NVivo 14 computer-aided qualitative data analysis software to aid analysis. Where possible any other relevant factors (e.g., the characteristics of the individuals in multidisciplinary teams and the settings they work in) will also be entered into NVivo.

Data analysis will be led by DS supported by other members of the team using the same realist logic of analysis that was used in previous studies [20,21]. A realist logic of analysis is a way of interrogating theory with data and of using theory to understand patterns in data to further refine the programme theory. A realist analysis of data follows a generative explanation for causation that is, an outcome (O) of interest was generated by relevant mechanism(s) (M) being triggered in context (C) [19]. These causal explanations are expressed as Context-Mechanism-Outcome-configurations (CMOcs). Data analysis is iterative over the course of the evaluation, with earlier stages of analysis being used to refine programme theory and/or refine evaluation design for subsequent stages. Following each data collection period, the analysis will involve reading and rereading the transcripts and other data sources before moving on to coding. In this sense the data is purposely mined for information that would help us test (confirm, refute or refine) the CMOcs identified in the realist review [21]. Where indicated by our interpretations of the data we will develop and refine new CMOcs based on the primary data collected in this study. This process is not linear and will involve discussion and deliberation between and across phases.

Throughout the analysis, the research team will move iteratively between the analysis of examples, refinement of programme theory, and further iterative primary data collection to test specific parts of the programme theory [21]. As mechanisms are often hidden or not articulated well, we will use retroductive reasoning to infer and elaborate on possible mechanisms. Retroductive analyses are analytical processes that seek to identify the hidden causal processes that lie beneath identified patterns or changes in those patterns [30]. Thus, our approach will involve repeatedly going from data to theory, to refine explanations about the occurrence of certain behaviours. We will discuss any conflicting interpretations of the data among the research team until a consensus is reached. We will endeavour to construct these explanations at a level of abstraction that would encompass a range of phenomena or patterns of behaviour. Where relevant we will draw on substantive theories to help us develop and test our emerging programme theory (e.g., Normalisation Process Theory [31]).

We will use any relevant quantitative data (e.g., audit data) that is available at each site to help us test our programme theory. For example, if a site has collected Patient Reported Outcome Measures (PROMS) data that shows HF patients are unhappy with the PC service, but service providers claim their service is excellent, then the PROMS data is useful for informing our interview guides in relation to asking patients what it is about the service that is not working for them, and similarly ask service providers why they think the service is excellent.

The refined programme theory and our findings will feed into Work package 2) and form the basis of our ‘how to guide’. The guide will detail our key findings on how the integration of PC and HF services produce their effects in various settings, along with a series of recommendations on how to tailor and implement integrated PC and HF services relating to each specific finding.

#### Work package 2: Co-design of a detailed ‘how to’ guide for setting up and improving integrated HF and PC services in the NHS.

We will use the programme theory from WP1 to co-design our detailed ‘how to’ guidance for setting up, sustaining and embedding the delivery of integrated HF and PC services. We will draw on behaviour change theory by Michie [28,32] to underpin the design of the ‘how to’ guidance. The behaviour change wheel (BCW) outlines nine intervention functions aimed at addressing deficits in a particular ‘behaviour system’. Our findings from WP1 will enable us to understand what behavioural changes are needed from integrated PC and HF service providers to optimise the delivery of their service. We will then use these findings to select the appropriate blend of interventions suggested by the BCW. The ‘how to’ guidance will include detailed information on how to use the ‘recommendations’ in different contexts and populations (i.e., different integrated PC and HF services).

Using a co-design approach [23,33] the ‘how to’ guidance will be developed through a series of focus groups with each of the five site teams. Focus groups will be led by DS and moderated by another member of the research team. Approximately 6–8 stakeholders will participate in the focus groups at each site (e.g., the integrated PC and HF service leads, PC and HF multidisciplinary teams n = 4–6, patients and informal carers n = 2). Focus groups will last up to 90 minutes, and involve an in-depth discussion of each recommendation in detail. The data generated will be subjected to a realist logic of analysis by the research team which involves identifying what outcome (O) of interest was generated by relevant mechanism(s) (M) being triggered in context (C), expressed as Context-Mechanism-Outcome-configurations (CMOcs). This analysis will inform the contents of the ‘how to’ guide. A draft of the ‘how to’ document will then be co-produced by the research team and the stakeholder group and shared with each site for review.

#### Work package 3: Develop a community of practice to support those who wish to set up, sustain and embed integrated HF and PC services.

Communities of practice are defined as groups of people with a common goal who regularly meet to provide support to each other, share and create knowledge together, and explore innovative ways to reach their goal [34,35]. Communities of practice are encouraged within health services and are well established in quality improvement efforts.

In this study, work packages 1 and 2 support the development of a co-designed ‘how to guide’ to help existing services deliver an integrated palliative care and heart failure approach. The community of practice in work package 3 will follow Wenger-Traynor’s model which has three core principles: setting up the community (who cares about the practice); identifying the domain (what is it we care about); and establishing the practice (what we do together to make it work) [35,36].

We will apply this model by:

1. Setting The Objective; we will ask our stakeholders who should be invited to the group and how best to approach them.
2. Create A Plan; Once identified, we will set up an initial meeting of those interested and collect each member’s goals and learning objectives. The plan will include a list of key resources required to facilitate learning outcomes within the group and other individuals/teams wanting to integrate their services.
3. Schedule Regular Meetings; we will organise up to three meetings (one per month) during WP3 using online platforms such as MS Teams or Zoom to facilitate a community of practice across the UK. WhatsApp will also be used to facilitate cross-site sharing.
4. Document the Process; we will video record the meetings (with attendees’ consent) and record the minutes so that shared knowledge is not lost. Documenting the meeting processes, personal lived experiences of integrating PC and HF, and subsequent discussions will help us accumulate shared learning and knowledge so we can keep track of progress and share learning with new members.

The community of practice will assist in the dissemination of findings among heart failure specialist clinics and provide support to those service providers who are interested in implementing an integrated model of care. The community of practice will allow us to assess if the ‘how to guide’ meets the needs of service providers and identify if there are facilitators or barriers to its implementation. In this way, the content of the community of practice meetings will help the research team to amend the ‘how to’ guide and ensure that it best meets the information and support needs of service providers. Sustainability of the community of practice will be facilitated by identifying champions for integrated PC and HF at each site who can maintain and build momentum after the project has been completed (33).

## Discussion

The provision of integrated PC and HF services is recommended across a range of guidelines and policies, but implementation has been slow and inconsistent. This study will use a realist evaluation approach to examine the implementation of integrated palliative care and heart failure services in the UK. The study will have a number of benefits. Firstly, direct improvement for delivery of five integrated PC and HF services in the UK. Secondly it will test and refine the programme theory of how integrated PC and HF services work, thereby providing more in-depth understanding of what works to set up, sustain and embed the delivery of integrated PC and HF services across the UK and beyond. This understanding will inform the development and design of a ‘how to’ guide that will help other service providers set up and sustain integrated PC and HF services. Thus, filling a current knowledge gap identified by PPI partners and providers. Thirdly, this study will result in improved integrated PC and HF services, with a network of experts (including the five sites) well-positioned to further advance knowledge and application of successful integrated services.

Findings from this study may also be relevant to other specialities who are seeking to improve integration and build capacity in their service provision, or those in other health service delivery models and jurisdictions. People with HF have multiple long-term conditions including HF and their PC need might arise from any of these (1). Therefore, there is a need to break down silos across specialities and settings (including geriatrics, primary and secondary and tertiary care settings) [37].

Given the practical and moral imperative of improving integrated care in a way that will improve access to PC for patients with HF, the outcomes of this project will be central to the NHS’s long term plan objective of providing more integrated services to address “increasing inequalities and pressures from a growing and ageing population” [38].

## Abbreviations

CMO: context mechanism outcome
COM-B: Capability Opportunity and Motivation – Behaviour
HCP: health care professional
HF: heart failure
MRC: medical research council
NI: Northern Ireland
NIHR: national institute for health and care research
PC: palliative care
PPI: patient and public involvement
PROMs: patient reported outcome measures
UK: United Kingdom
USA: United States of America
WP: work package.
